# Flexibility of Expressive Timing in Repeated Musical Performances

**DOI:** 10.3389/fpsyg.2016.01490

**Published:** 2016-10-04

**Authors:** Alexander P. Demos, Tânia Lisboa, Roger Chaffin

**Affiliations:** ^1^Department of Psychology, University of Illinois at Chicago, ChicagoIL, USA; ^2^Centre for Performance Science, Royal College of MusicLondon, UK; ^3^Department of Psychology, University of Connecticut, StorrsCT, USA

**Keywords:** performance, expression, musical gesture, expressive timing, practice

## Abstract

Performances by soloists in the Western classical tradition are normally highly prepared, yet must sound fresh and spontaneous. How do musicians manage this? We tested the hypothesis that they achieve the necessary spontaneity by varying the musical gestures that express their interpretation of a piece. We examined the tempo arches produced by final slowing at the ends of phrases in performances of J. S. Bach’s No. 6 (Prelude) for solo cello (12 performances) and the Italian Concerto (Presto) for solo piano (eight performances). The performances were given by two experienced concert soloists during a short time period (3½ months for the Prelude, 2 weeks for the Presto) after completing their preparations for public performance. We measured the tempo of each bar or half-bar, and the stability of tempo across performances (difference of the tempo of each bar/half bar from each of the other performances). There were phrase arches for both tempo and stability with slower, less stable tempi at beginnings and ends of phrases and faster, more stable tempi mid-phrase. The effects of practice were complex. Tempo decreased overall with practice, while stability increased in some bars and decreased in others. One effect of practice may be to imbue well-learned, automatic motor sequences with freshness and spontaneity through cognitive control at phrase boundaries where slower tempi and decreased stability provide opportunities for slower cognitive processes to modulate rapid automatic motor sequences.

## Introduction

In order to be reliable under the pressures of the concert stage, musical performances by concert soloists in the Western classical tradition are prepared and practiced until they become thoroughly automatic (Chaffin and Imreh, 2002). At the same time, these performances should sound fresh and spontaneous (Chaffin et al., 2007). As the Russian pianist Emil Gilels notes, “*When I am in top form... the ideas are always different. Sometimes I play with greater changes in dynamics, sometimes with less... I must say it is different each time I play, and it is a process which… includes mastery of the work, knowing the details, being comfortable with it, and then adding the fantasy*” (Emile Gilels, in Mach, 1991, p. 123). Gilels clearly believes that his ability to be spontaneous is a reflection of both his artistry and his thorough preparation.

Gilels appears to contradict the assumption of *dual process theories* of skill learning that highly practiced skills are automatic (i.e., performed rapidly, with minimal variation, without attention or intention, with little conscious awareness, and without interfering with other activities) and are controlled only intermittently by slower, more deliberative, conscious thought processes (Fitts and Posner, 1967; Posner and Snyder, 1975; Shiffrin and Schneider, 1977; Dreyfus and Dreyfus, 1986; see Evans and Stanovich, 2013 for a review). Instead, Gilels seems to describe an ongoing interweaving of automatic and controlled processes of the sort proposed by *systems theories* that emphasize the role of the evolving situation in shaping the execution of automatic routines (Endsley, 1995; Miller, 2000; Duncan, 2010; see Christensen et al., 2016 for a review). Most dual process theories of skill learning allow for some intermittent cognitive control of automatic lower level skills to account for the ability of expert performers to select strategies, change styles, and engage in problem solving. In contrast, systems theories propose that controlled and automatic processes are continuously interwoven in most skills (Ericsson, 2006; Schmidt and Wrisberg, 2008; Evans and Stanovich, 2013; Christensen et al., 2016).

We explored these issues by examining the stability of tempo across repeated performances by an experienced concert soloist (the second author) of a piece that had been thoroughly prepared for public performance. Gilels does not indicate at what points, during a performance, he takes cognitive control. We looked at boundaries between phrases, as a likely candidate. Performers use phrase arches to communicate their musical interpretations to listeners, continuously varying tempo and loudness across phrases so as to delineate their beginnings and ends (Clarke, 1989). Phrase arches have been documented for a wide variety of pieces in the Western art music canon, and for musicians of varying levels of expertise (see Gabrielsson, 1999, 2003 for reviews; Shaffer, 1981; Todd, 1985; Repp, 1997b). Performers generally agree on the overall shape of the phrasing profile for a piece, while also differing consistently from one another in ways that reflect each musician’s unique interpretation (Repp, 1995). Performers signal to audiences the importance they assign to different phrase boundaries by varying the size and shape of phrase arches (Dodson, 2011). These interpretive nuances are often replicated across performances with remarkable precision, and are more pronounced for professional than for student musicians, befitting their status as reflections of musicianship and artistry (Repp, 1995, 1997a,b).

Phrase arches can be described by quadratic equations (Todd, 1985; Shaffer and Todd, 1987), expressive performance rules (Friberg et al., 2006), or computationally (Widmer and Tobudic, 2003; Widmer and Goebl, 2004). However, there is little agreement as to their source (Thompson, 2009, p. 191). Their similarity to the temporal profile of other activities such as locomotion (Friberg and Sundberg, 1999) and speech (Tan et al., 2010, p. 213), suggests that phrase arches are produced by basic grouping processes involved in perception, memory retrieval, or motor planning (Palmer and Pfordresher, 2003; Honing, 2005; Palmer, 2005; van Vugt et al., 2012). Whatever their source, phrases are a basic temporal unit of musical performance and are, thus, a good place to look for evidence of the differences between performances that Gilels refers to as “fantasy.”

If Gilels is right that differences between performances are the result of “adding the fantasy,” then increased cognitive control should be accompanied by increased differences between performances. To test this prediction, we measured the stability of tempo across multiple performances of the same piece, separately for each bar. We hypothesized that automatic playing would result in more stability, i.e., more similarity between performances, and that cognitive control would result in less stability, i.e., less similarity between performances. We expected cognitive control to increase at phrase boundaries because this is where slower tempi most consistently provide opportunities for slower cognitive processes to modulate rapid automatic motor sequences. Therefore, we expected to find phrase arches for stability similar those for tempo and loudness: lower stability at phrase boundaries, reflecting increased cognitive control; higher stability mid-phrase, reflecting greater automaticity of action sequences once the phrase is launched.

Every theory of skill learning maintains that fluent execution of complex motor skills requires automaticity and that automaticity requires practice (Christensen et al., 2016). This appears to predict that both tempo and stability should increase with practice. There is some evidence that this does happen early on in practice as hesitations disappear and playing becomes fluent (Chaffin et al., 2006, 2007). However, the performances that we studied were long past this stage. These were polished, public performances. In Gilels’ terms, they were past the stage of achieving “mastery of the work”; the performers were ready for “adding the fantasy.” We had recorded the entirety of the practice leading up to the performances and so were able to include the amount of practice received by each bar as a predictor. If Gilels is correct, then passages that receive more practice will be less stable. If traditional theories of skilled action are correct, then the relationship will be the reverse: passages that are practiced more will be more stable (Dreyfus and Dreyfus; Fitts and Posner, 1967; Posner and Snyder, 1975; Shiffrin and Schneider, 1977).

We examined the stability of tempo across multiple performances of the Prelude of J. S. Bach’s Suite No. 6 for solo cello. We had recorded the preparation of the Prelude for public performance over a period of 22 months as part of an earlier longitudinal study of expert practice (Chaffin et al., 2010). In the present study, we examined 12 performances from the last 3½ months of this study during the time when the cellist was performing the Prelude in public. We examined five public performances and the seven practice performances that occurred during practice sessions that were interspersed between the public performances. After completing the analyses of the Prelude, we performed the same analyses on a similar set of eight polished performances of the third movement (Presto) of J. S. Bach’s Italian Concerto, recorded during the last 2 weeks of a 10-month longitudinal study of expert practice (Chaffin and Imreh, 2002; Chaffin et al., 2002, 2007).

The same performances of the Presto were the focus of a previous study that examined similarities and differences between repeated performances (Chaffin and Logan, 2006; Chaffin et al., 2007). The earlier study took a different approach, examining differences between performances in the musical gestures associated with musical motifs that were repeated multiple times throughout the piece. For example, the main theme of the Presto, which repeats six times, is introduced by a downward octave jump. This musical motif was accentuated on each of its six repetitions by prolongation of the preceding note, which had the effect of emphasizing the downward jump. This gesture was significantly larger in one performance and significantly smaller in a second performance, compared to the mean across all performances. There were significant differences of this sort for four of the nine musical gestures examined, providing empirical support for Gilels’ suggestion that well-prepared performances differ in musically meaningful ways. The present approach to measuring stability has the advantage over the earlier method that it applies equally to every bar, rather than being limited to specific musical gestures, and does not require comparison with a mean performance, because comparison is built into the metric.

The Prelude and Presto are similar in length, composer, and stature as important works in their respective repertoires. Both require a high degree of automaticity of the performer. In other respects, they are very different. The Prelude, often described as “exuberant,” is written in a “quasi-improvisatory character” that progresses through the circle of fifths, using bariolage (string crossings) for dramatic effect, and spanning both the low and high registers of the instrument (Winold, 2007). Written for the viola pomposa, an instrument with five strings, the Prelude is a virtuoso piece when played on the four strings of the modern cello, requiring many large and rapid left-hand position changes on the fingerboard (Chaffin et al., 2010; Lisboa et al., 2011). For the Presto, the main challenge is the fast tempo and perpetuum mobile (without rest) style which provide no opportunity for performers to gather their thoughts about what comes next, making it hard to keep track of the many small variations in the three different themes, each of which repeats multiple times (Chaffin et al., 2002, pp. 94–97). We were interested to see whether, despite their differences, stability of tempo across performances would behave in similar ways in both pieces.

## Materials and Methods

### Music and Musicians

The Prelude from J. S. Bach’s Suite No. 6 for solo cello is notated in 12/8 time in 104 bars, and takes about 5 min to perform. For analysis, we divided the bars into 208 half-bars, which we refer to below as “bars.” The Presto of the Italian Concerto by J. S. Bach is notated in 210 bars in 2/2 time, lasts 3–4 min, and is of moderate technical difficulty. The cellist, Tânia Lisboa (the second author), was trained as a concert pianist and cellist in her native Brazil before continuing her study of the cello in England and France. The pianist, Gabriela Imreh, was trained in classical piano in Romania before moving to the US. Both musicians perform regularly as soloists. The musicians were unaware of the hypotheses about stability which were formulated after the longitudinal studies had ended.

### Performances and Practice

We examined 12 performances of the Prelude from a 3½ month period at the end of a longitudinal study lasting 22 months during which the cellist recorded 32 out of 38 h of practice and all public performances (Chaffin et al., 2010; Lisboa et al., 2011). We examined five public performances and seven practice performances that occurred during practice sessions interspersed between the live performances. Three additional public performances were not included, the first because the cellist was less satisfied with it than with the other performances, and the second and sixth due to recording errors.

We examined eight performances of the Presto, seven practice performances and the final performance released on CD (Imreh, 2009). All performances took place during a 10-day period at the end of a longitudinal study lasting 10 months during which the pianist recorded 28 out of 33 h of practice (Chaffin and Imreh, 2002; Chaffin et al., 2002). One public performance, partway through the learning process, was not recorded (Chaffin et al., 2002, pp. 110–111).

We measured the amount of practice for each bar by counting frequency of times the performer started practice on that bar. We excluded the first bar of each piece. The frequency distributions were positively skewed (*M* = 23.01, *SD* = 30.32, range = 0–206; *M* = 23.30, *SD* = 33.24, range = 0–171, for the Prelude and Presto respectively).

### Reports and Predictors

As part of the earlier longitudinal studies, the two musicians provided reports of the musical structure, marking copies of the score to indicate the location of sections and sub-sections (Chaffin et al., 2002, 2010, pp. 171–178 for the Prelude and Presto respectively). In the present study, we used the reports of sub-sections, which we refer to below as “phrases” for consistency with other studies of phrase arches. The musicians identified 44 phrases for the Prelude and 37 phrases for the Presto. We coded location within phrases as serial position from the beginning of each phrase.

### Measurement of Tempo and Stability

For each performance of each piece, we used SoundForge, a commercial sound wave processing program, to measure inter-bar-intervals (IBI, in seconds) from the start of the first note sounded in each bar to the start of the first note of the next. Then we converted IBIs to tempo measured in beats per minute [tempo = (1/IBI in seconds) × # beats per bar × 60 s/min]. We removed the first and last bar of each piece from all analyses as those bars may behave differently for a variety of reasons and removing them was conservative with respect to finding phrase arches.

We measured stability for each bar in each performance by first subtracting the tempo for each bar from the tempo of the previous bar to calculate the tempo change for each bar in each performance. Next we subtracted the tempo change of each target bar from the tempo change of the same bar in each of the other performances, averaging the absolute values to measure how different the tempo change of that bar was from the tempo change of the same bar in all the other performances. These values were then flipped to create stability scores and linearly rescaled by subtracting from each bar the maximum stability value of all the bars in all the performances (max), multiplying by -1, and finally rescaling the values to between 0 and 1 by dividing by max. Thus, “1” means complete stability, i.e., the same tempo in each performance, and “0” means minimal stability, i.e., maximal difference in tempo from each of the other performances. A sample calculation is provided in the Supplementary Materials along with a comparison between this stability metric and the standard deviation.

This stability metric differs from alternatives in comparing each performance directly to each other performance rather than to a mean or expected performance. Other stability metrics assess stability by computing deviations from an expected value (van Vugt et al., 2012) or mean performance (Repp, 1997c), or by regressing out the mean performance (Chaffin et al., 2007). The different metrics correspond to different assumptions about the underlying processes involved. Comparison with a mean or expected value corresponds to the widely held view that behavior is the product of underlying motor programs to which the implementation process adds noise (e.g., van Vugt et al., 2012). Our metric avoids this assumption and, instead, takes Gilels’ description of performance as “different each time” at face value by treating each performance as a unique event. Thus, our metric is appropriate for the claim that we evaluated. The simulation research needed to fully ascertain the properties of the different metrics is beyond the scope of this inquiry. However, we note that our metric is more versatile than that used by Chaffin et al. (2007) which is limited to musical gestures that are repeated multiple times within the same piece, e.g., prolongation of the downward octave jump, and requires comparison with a mean performance. In contrast, the current metric measures stability for every bar and does not require comparison with a mean performance because this is built in to the metric. Significant effects will indicate that stability across performances changed reliably, e.g., as a function of position in a phrase.

### Analysis

We used mixed effect models to examine the effects of phrasing and practice (Singer and Willett, 2003), using the LME4 package in R (Bates et al., 2015). We analyzed tempo and stability separately, using the same two models for each. The first model included as fixed effects: serial position within a phrase (linear, and quadratic effects), and, for the Prelude only, performance type (live or practice, dummy coded “0” and “1” respectively). The second model added amount of practice measured by number of starts (in z-scores) of each bar (linear, quadratic, cubic, and quartic effects). We included as random effects the intercepts for performances and serial position (slopes) within a phrase (linear and quadratic effects). This controlled for differences between performances and between phrases within a performance, making it easier to detect fixed effects of serial position and practice. To test if the addition of practice significantly improved the model fit, a deviance test was conducted (Singer and Willett, 2003). The significance of individual predictors was tested using t-values assessed as if they were Z-values (Barr et al., 2013).

## Results

**FIGURE 1:**
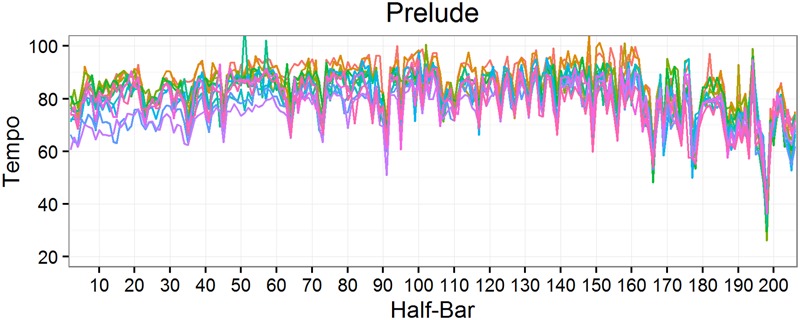
**Tempo of the 12 performances of the *Prelude***.

**FIGURE 2:**
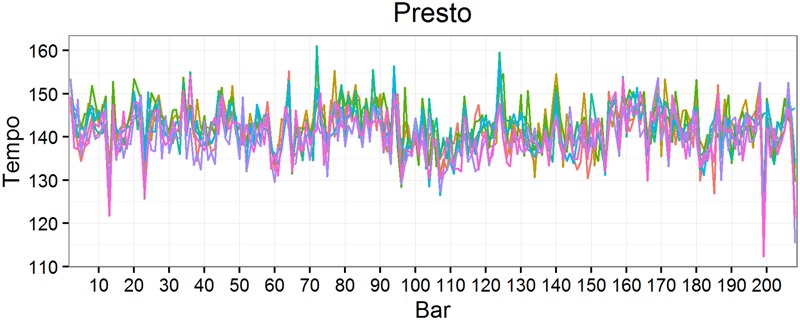
**Tempo of the eigth performances of the *Presto***.

**Figures 1** and **2** show tempo for each performance of the Prelude and Presto respectively. Where the tempi for each performance align, there is high stability, e.g., just before half-bar 200 of the Prelude, where tempi drop to about 30 bpm (**Figure 1**), and just after bar 120 of the Presto, where tempi rise above 150 bpm (**Figure 2**). Visual inspection suggests that tempo and stability were relatively independent, an impression confirmed by their low mean correlation (*r*) within performances for both the Prelude, *M* = 0.146, *SD* = 0.115 and Presto, *M* = 0.004, *SD* = 0.081.

### Tempo

**FIGURE 3:**
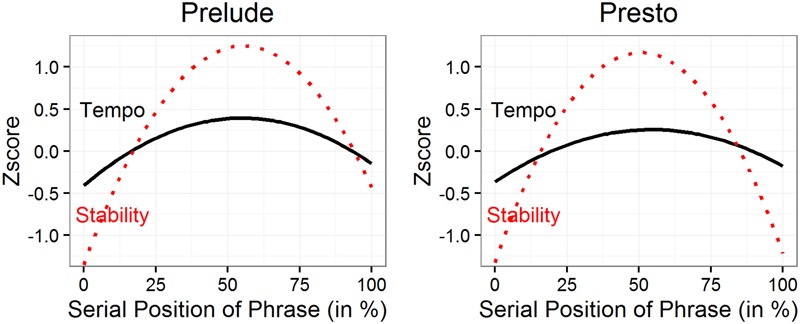
**Tempo and stability as a function of serial position in a phrase (based on Model 1)**.

The mixed models for tempo are summarized in **Table 1**. Effects of phrasing are shown in **Figure 3** for both tempo and stability. For tempo, there were arch-shaped serial position functions for both pieces, with slower tempi at beginnings and ends of phrases and faster tempi in the middle, with peaks just after the halfway mark. This was reflected in significant quadratic effects of phrasing in Model 1, for both pieces. For the Prelude, there was also a marginally significant positive linear effect in Model 1, indicating that playing was faster at beginnings than at ends of phrases. This effect disappeared when practice was added in Model 2, suggesting that it was a product of the differing amounts of practice received by different bars. The quadratic effect of phrasing was not affected by the inclusion of practice in Model 2, indicating that the tempo arches were not a product of differences in the amount of practice received by different serial positions in a phrase.

**Table 1:** Mixed effects models for the Prelude and Presto: tempo.

	Prelude		Presto	
Fixed effects	Model T1	Model T2	Model T1	Model T2
Intercept	78.715^∗∗∗^	78.629^∗∗∗^	142.128^∗∗∗^	142.097^∗∗∗^
	(1.190)	(1.201)	(0.604)	(0.602)
Live	5.291^∗∗∗^	5.308^∗∗∗^	-	-
	(1.233)	(1.249)	-	-
Phrase	42.070^†^	1.527	2.162	0.713
	(22.737)	(23.853)	(12.003)	(12.570)
Phrase^2^	-109.960^∗∗∗^	-79.058^∗∗∗^	-37.630^∗∗^	-37.486^∗∗^
	(14.058)	(14.394)	(12.376)	(12.579)
Practice		-91.552^∗∗∗^		-5.742
		(11.067)		(7.435)
Practice^2^		-2.077		-1.010
		(13.033)		(6.823)
Practice^3^		-41.609^∗∗∗^		-17.621^∗∗^
		(9.303)		(6.513)
Practice^4^		32.885^∗∗∗^		-4.285
		(6.955)		(6.160)

**Random effects**				

Phrase (intercept)	40.393	40.522	5.900	5.835
Performance (intercept)	4.315	4.477	1.345	1.345
Phrase	25576.196	27334.499	4687.092	5056.482
Phrase^2^	6754.285	6807.010	5036.750	4859.816
Residual	22.712	19.260	15.609	15.482

**Goodness of fit**				

Log likelihood	-7583.785	-7518.326	-4185.370	-4180.643

*^†^*p* < 0.1, ^∗∗^*p* < 0.01, ^∗∗∗^*p* < 0.001.*

**FIGURE 4:**
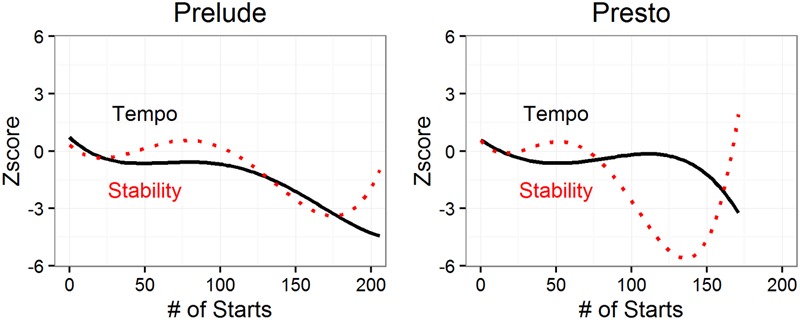
**Tempo and stability as a function of amount of practice (number of starts)**.

The addition of practice in Model 2 improved model fit for both the Prelude, χ^2^(4) = 130.92, *p* < 0.0001, and the Presto, χ^2^(4) = 9.454, *p* = 0.0507. The effects of practice are shown in **Figure 4**, for both tempo and stability. The complex practice functions were strikingly similar for the two pieces. Tempo was faster for bars that received the least practice (probably in the middle of phrases) and slower for bars that received the most practice, and plateaued for bars that received intermediate amounts of practice. When we measured amount of practice by the number of repetitions (instead of number of starts), results were similar. For the Prelude, the linear, cubic, and quartic terms were significant, while for the Presto only the cubic term was significant. Despite these differences in the significance of the various terms, we modeled practice in the same way for both pieces because of striking similarity of the complex practice functions for the two pieces (see **Figure 4**). The differences between the two pieces in the significance of the various terms is mostly a reflection of differences for bars that received the most practice, resulting in a leveling of the downward trajectory for the Prelude but not for the Presto.

### Stability

The mixed models for stability are summarized in **Table 2**. In **Figure 3** there are pronounced arches for stability for both pieces, with peaks occurring just after the half way mark, similar to those for tempo. Thus, stability was lowest (different tempi) at the starts and ends of phrases, and maximal (same tempi) in the middles of phrases. The arched shaped functions were reflected in significant quadratic effects in Model 1, for both the Prelude and Presto. The inclusion of practice in Model 2 did not change the quadratic phrase effect for the Prelude, but did reduce it for the Presto, suggesting that, for this piece, stability arches were partly due to differential practice of different serial positions within a phrase.

**Table 2:** Mixed effects models for the Prelude and Presto: stability.

	Prelude		Presto	
Fixed effects	Model S1	Model S2	Model S1	Model S2
Intercept	86.088^∗∗∗^	85.727^∗∗∗^	81.511^∗∗∗^	81.453^∗∗∗^
	(0.902)	(0.996)	(0.822)	(0.869)
Live	-0.524	-0.516	–	–
	(0.813)	(0.812)	–	–
Phrase	21.307	41.143^⋅^	1.697	-9.891
	(20.421)	(21.602)	(29.277)	(29.005)
Phrase^2^	-56.380^∗∗^	-69.575^∗∗∗^	-59.907^∗∗^	-39.481^†^
	(18.691)	(20.029)	(22.419)	(22.394)
Practice		1.414^∗∗^		-39.991^∗^
		(0.549)		(17.616)
Practice^2^		1.327		-1.446
		(0.828)		(16.130)
Practice^3^		-0.815^∗^		31.909^∗^
		(0.346)		(15.360)
Practice^4^		0.092^∗^		51.702^∗∗∗^
		(0.036)		(14.755)

**Random effects**				

Phrase (intercept)	26.641	27.249	18.484	21.332
Performance (intercept)	1.479	1.480	0.697	0.708
Phrase	16706.898	15898.269	27778.378	26423.034
Phrase^2^	9888.691	10602.652	14764.486	12739.349
Residual	91.550	91.090	95.111	93.800

**Goodness of fit**				

Log likelihood	-9178.043	-9172.521	-5467.098	-5457.226

*^†^*p* < 0.1, ^∗^*p* < 0.05, ^∗∗^*p* < 0.01, ^∗∗∗^*p* < 0.001.*

The addition of practice improved model fit for both the Prelude, χ^2^(4) = 11.044, *p* = 0.0261, and the Presto, χ^2^(4) = 19.743, *p* < 0.0001. The W-shaped effect of practice in **Figure 4** was strikingly similar for the two pieces. The complexity of its shape was reflected in significant linear, cubic and quartic terms for both the Prelude and Presto. As with tempo, the effects were similar when we measured practice by repetitions instead of starts. Stability decreased with practice for bars that received very little practice (<20 starts), and for bars that received a lot of practice (75–130 starts). Stability increased with practice for bars that received intermediate levels of practice (20–60 starts) and again for the small number of bars that received the most practice. The upturns at the upper end of the curves were significant for both pieces, despite the small number of bars involved (*N* = 2 and 4 for the Prelude and Presto, respectively, times the number of performances), as indicated by the significant quartic terms in Model 2. These bars were all at beginnings of phrases and were thus exceptions to the overall relationship between phrasing and stability evident in **Figure 3**.

## Discussion

Repeated performances of the same piece differed in musically meaningful ways, as Emile Gilels suggested. The stability of tempo across multiple, polished performances was systematically related to musical structure: higher in mid-phrase, lower at beginnings and ends. We found phrase arches for stability in two pieces of very different musical character, played on different instruments by different performers, suggesting that stability arches are a general characteristic of musical performance. Stability decreased at phrase boundaries, suggesting that this is where the musicians added “fantasy” to their performances by using cognitive control to moderate the activity of less flexible automatic processes.

We also found tempo arches similar to those reported in previous studies (Gabrielsson, 1999, 2003). Unlike earlier studies, which examined specific phrases (Repp, 1995, 1997b), we examined entire pieces. This was made possible by our use of mixed-effects models, which allowed us to estimate the reliability of tempo (and stability) arches across phrases, while statistically controlling differences in musical material across phrases within each piece. We could have also examined the two pieces in the same statistical model. We chose to analyze them separately because the Presto data provided a replication for the Prelude study.

Tempo and stability followed similar trajectories across the course of a phrase. This is not because they measured the same thing. Overall, the correlation between tempo and stability was low. Tempo and stability arches do not always coincide. For example, when pianists play two-octave scales, there are separate stability arches for each octave but only a single tempo arch tying the two octaves together (van Vugt et al., 2012). This suggests that stability arches reflect transitions between well-learned motor sequences. In our study, phrasing and motor sequences coincided because practice was organized in terms of musical structure, starting and stopping at phrase boundaries (Chaffin and Imreh, 2002; Chaffin et al., 2010). As a result, motor sequences and phrases were aligned.

The likelihood that arches for stability and tempo reflect transitions between well-learned motor sequences does not preclude the possibility that they are also occasions for “fantasy,” in Gilels’ terms, or the interaction of controlled and automatic processes, in our terms. Rather, it seems likely that it is precisely the slower tempi at transitions between stored motor sequences that makes phrase boundaries the natural location for musicians to think about what to do next (add fantasy). It seems likely that slowing at phrase boundaries serves multiple purposes: transitioning to a new motor sequence (van Vugt et al., 2012), modulating the components of well-practiced motor sequences, and highlighting the musical structure for the listener (Clarke, 1989). We examined only one aspect of the process, showing that differences between performances are largest at phrase boundaries. Further research is needed to understand how this between-performance variability is related to properties of individual performances and to listeners’ perceptions of them. Fuller understanding of how performers communicate their musical intentions to audiences will require examination of the interaction between the multiple components (listener, performer, and setting) of a complex system (Hargreaves et al., 2005; Sawyer, 2005).

Our data do not allow us to say whether slowing was restricted to boundaries between phrases, as predicted by dual process theories of motor skill, or whether cognitive and automatic processes were more continuously interwoven throughout each phrase, as predicted by systems theories (Evans and Stanovich, 2013; Christensen et al., 2016). The smooth continuity of the stability functions suggests continuous interweaving, but it is equally possible that cognitive intervention occurred at boundaries between phrases and that the smoothness of the stability function was a product of averaging. In either case, the stability arches suggest that cognitive control occurred frequently, increasing at phrase boundaries and decreasing in mid-phrase.

There was a complex relationship between stability and practice. For some bars, stability decreased with practice, supporting Gilels’ claim that practice is required to engage in “fantasy,” but only for bars that received very little practice (<20 starts) or a lot of practice (75–130 starts). At intermediate levels of practice (20–60 starts), stability increased with practice, as predicted by theories of motor skill learning (Dreyfus and Dreyfus; Fitts and Posner, 1967; Posner and Snyder, 1975; Shiffrin and Schneider, 1977). Stability was also higher for the very small number of bars that received the most practice. These bars appeared to be expressive highpoints that were also difficult technically. Since practice was not controlled experimentally, we cannot be sure of the explanation for its complex effects. However, the striking similarity of the effects in the two pieces suggests that they were not artifactual and merit further study.

Comparing multiple performances of the same piece provides a window into the processes responsible for the complex motor and cognitive skills involved in performance. Our discovery that tempo and stability arches coincide in well-prepared musical performances deepens our understanding of tempo arches, helping to understand how musical expression is generated (Clarke, 1989). Our results verify the last part of Gillels’ claim that, “*When I am in top form... the ideas are always different… It is different each time I play,”* and show that the differences are centered on phrase boundaries. If Gilels is right about the rest of his claim, then differences between performances at phrase boundaries may reflect the interplay between controlled and automatic processes as the performer adjusts the performance to musical ideas for the upcoming phrase.

## Author Contributions

RC and TL collected the data for this project. AD, TL, and RC worked on various drafts of the manuscript. AD created the analysis and AD, TL, and RC interpreted the results. All authors agree to all aspects of this paper.

## Conflict of Interest Statement

The authors declare that the research was conducted in the absence of any commercial or financial relationships that could be construed as a potential conflict of interest.
