# Supplementary material for: Flexibility of Expressive Timing in Repeated Musical Performances
Source: Front Psychol. 2016 Oct 4;7:1490. doi: 10.3389/fpsyg.2016.01490 (PMC5047881; doi:10.3389/fpsyg.2016.01490)
Supplement: Supplementary file 1 [file Data_Sheet_1.DOCX]

Supplementary Material

Flexibility of Expressive Timing in Repeated Musical Performances

Alexander P. Demos^1*^, Tânia Lisboa^2^, Roger Chaffin^,3^

*** Correspondence:** ademos@uic.edu

# Supplementary Methods

The following example shows how we calculated stability and how our metric differs from the standard deviation (SD).

## Stability Calculation.

Table 1 shows bar-to-bar tempi for five performances of a five bar phrase.

Table 1. Raw tempo for five performances.

| Bar | P1 | P2 | P3 | P4 | P5 | Mean | SD | CV |
| --- | --- | --- | --- | --- | --- | --- | --- | --- |
| 1 | 60 | 55 | 62 | 64 | 50 | 58.2 | 5.67 | 0.098 |
| 2 | 57 | 50 | 56 | 60 | 20 | 48.6 | 16.40 | 0.337 |
| 3 | 62 | 61 | 64 | 63 | 62 | 62.4 | 1.14 | 0.018 |
| 4 | 59 | 58 | 57 | 59 | 60 | 58.6 | 1.14 | 0.019 |
| 5 | 50 | 54 | 53 | 51 | 52 | 52 | 1.58 | 0.030 |

Table 2 shows the absolute values of the within performance difference (delta) scores, i.e., tempo change.

Table 2. Tempo change: absolute value difference (delta) scores within performances

| Bar | P1 | P2 | P3 | P4 | P5 | Mean | SD | CV |
| --- | --- | --- | --- | --- | --- | --- | --- | --- |
| 2-1 | 3 | 5 | 6 | 4 | 30 | 9.6 | 11.46 | 1.194 |
| 3-2 | 5 | 11 | 8 | 3 | 32 | 11.8 | 11.69 | 0.991 |
| 4-3 | 3 | 3 | 7 | 4 | 2 | 3.8 | 1.92 | 0.506 |
| 5-4 | 9 | 4 | 4 | 8 | 8 | 6.6 | 2.41 | 0.365 |

Table 3 illustrates the next step, which is to calculate a matrix of the absolute differences in tempo change between all of the performances. This is done bar-by-bar, for each pair of performances. So, in Table 3, we show calculations for a single bar, Bar 2-1. Similar matrices are computed for each bar. As the matrix is symmetrical, we can ignore one side of the major diagonal. The mean for each row provides the mean of the absolute differences for that performance, summarizing how much it differs from each of the other performances. Each mean is a normalized measure of (in)stability. Thus, we can see in Table 3 that Performances 1 - 4 are fairly similar to one another, whereas Performance 5 is different. The grand mean for the five performances (M = 11.2) is similar, but not identical, to the standard deviation (SD = 11.46) of the raw difference scores (3, 5, 6, 4, 30) for Bar 2-1. We will explore the differences between our method and the SD after we explain the normalization procedure, which requires that we examine a series of bars.

Table 3. Difference in tempo change: Matrix of absolute value difference (delta) scores between performances, for Bar 2-1.

| Change in  Tempo | P1 (=3) | P2 (=5) | P3 (=6) | P4 (=4) | P5 (=30) | Mean |
| --- | --- | --- | --- | --- | --- | --- |
| P1 (=3) |  | 2 | 3 | 1 | 27 | 8.25 |
| P2 (=5) | 2 |  | 1 | 1 | 25 | 7.25 |
| P3 (=6) | 3 | 1 |  | 2 | 24 | 7.5 |
| P4 (=4) | 1 | 1 | 2 |  | 26 | 7.5 |
| P5 (=30) | 27 | 25 | 24 | 26 |  | 25.5 |

Table 4 shows the (in)stability values for each bar in each performance. The first row contains the values from Table 3.

Table 4. (In)Stability scores.

| Bar | P1 | P2 | P3 | P4 | P5 | Mean | SD | CV |
| --- | --- | --- | --- | --- | --- | --- | --- | --- |
| 2-1 | 8.25 | 7.25 | 7.5 | 7.5 | 25.5 | 11.2 | 8.00 | 0.715 |
| 3-2 | 12 | 12 | 11.25 | 13.5 | 35.25 | 16.8 | 10.35 | 0.616 |
| 4-3 | 1.5 | 1.5 | 4 | 1.75 | 2.25 | 2.2 | 1.05 | 0.478 |
| 5-4 | 3 | 3.25 | 3.25 | 2.25 | 2.25 | 2.8 | 0.51 | 0.183 |

Table 5 shows the same values converted to stability scores by flipping them to create stability scores for which 0 represent maximal instability and 1 = maximal stability, and then normalizing them. First, we flip them: stability score = [each instability score - max(of all instability scores) * -1]. Next, we normalize them: stability score / max (of all stability scores).

Table 5. Stability scores (normalized).

| Bar | P1 | P2 | P3 | P4 | P5 | Mean | SD | CV |
| --- | --- | --- | --- | --- | --- | --- | --- | --- |
| 2-1 | 0.80 | 0.83 | 0.82 | 0.82 | 0.29 | 0.71 | 0.24 | 0.333 |
| 3-2 | 0.69 | 0.69 | 0.71 | 0.64 | 0.00 | 0.55 | 0.31 | 0.561 |
| 4-3 | 1.00 | 1.00 | 0.93 | 0.99 | 0.98 | 0.98 | 0.03 | 0.032 |
| 5-4 | 0.96 | 0.95 | 0.95 | 0.98 | 0.98 | 0.96 | 0.02 | 0.016 |

In any statistical analysis of tempo, the reliability of the statistical inference decreases as variability between performance increases. Stability handles this variability differently from other measures that require computing deviation from a mean, such as the SD. For example, in Table 2, bars 2-1 and 3-2 both have critical values (CV = SD/mean) of close to 1. A t-test comparing bars 2-1 and 3-2 shows no difference, paired-*t*(4)=-1.87, *p* = .13, indicating that the mean of 9.6 bpm is not different from 13.8. In contrast, in Table 5 the same bars have means of .71 vs .55, which are significantly different, paired-*t*(4)=5.03, *p* = .007, indicating that bar 2-1 is more stable than bar 3-2. Given what we see in Table 2, this is clearly the preferred conclusion.

## Stability vs standard deviation with outliers.

Below, we compare our measure of stability to the standard deviation. We summarize the simulation of 1000 trials for 2 bars and 12 performances. Bar 1 was set to be randomly normally distributed with a tempo of 80 bpm with an SD = 4 bpm. Bar 2 was set to be faster, relative to Bar 1, with a randomly normally distributed increase in tempo of 10 bpm, with an SD = 4 bpm. Performance 12, Bar 2 was set to be an outlier, with a randomly normally distributed increase in tempo of 30 bpm, with an SD = 4 bpm. We calculated two dependent variables: our stability metric and the standard deviation of change of tempo. We did not use our normalization procedure for our metric of stability for these tests so that both SD and stability were in the same units (beats per minute). We then calculated a regression analysis with the independent variable being the degree of “outlierness” of the 12^th^ performance [Performance 12 change in tempo – mean change in tempo for Performances 1 - 11]. The steeper the slope of the regression line, the more impact the outliers have on the metric of stability. As can be seen in Figure 1, the two metrics respond differently to outliers. The slope appears shallower for stability than for the SD measure due to the higher intercept and less compressed range.

| 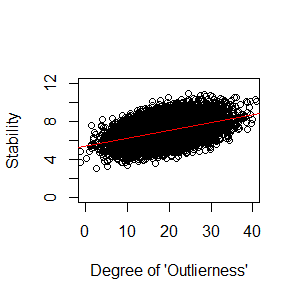 | 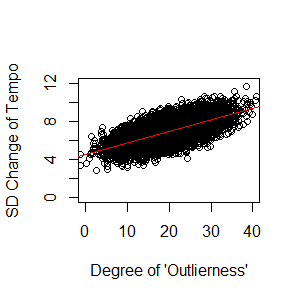 |
| --- | --- |

Figure 1. Simulation of 1000 trials for Stability and SD of change of tempo.

Next, we compared the slopes for the two measures by bootstrapping. We calculated the slope from the regression 100 times for each measure to provide an estimate of the mean and standard error. We summarize the results in Table 6. We next conducted boot-*t* tests on the mean slopes to compare our method with the SD method (Efron & Tibshirani, 1993). The t-test shows our method has a significantly shallower slope, boot-*t =* -117.25*, p <* .0001. This suggests that our measure is more robust in the case of outliers. However, this may only be the case when there are a sufficient number of performances. Further simulations are needed to assess the impact of number of performances, variability, autocorrelation between bars, as well as other factors.

Table 6. Results of bootstrapping stability metrics.

| **Metric** | **Mean Slope** | **SEM** |
| --- | --- | --- |
| Stability | .0846 | .00017 |
| SD change in tempo | .1214 | .00015 |

Finally, to estimate the similarity between the two measures, we bootstrapped an R^2^ (using linear regression) following the same procedures described above. The two measures are very similar (as they should be), but sufficiently different to suggest that they measure somewhat different aspects of stability across performances, R^2^ =.788, SE = .040.

## Advantages of stability

Our stability metric has an important advantage over methods based on computing the mean, such as the SD (Repp, 1995) or interquartile range (Van Vugt et al, 2012) when used in combination with mixed model regression. Our stability metric retains information about the stability of each bar and each performance. Mixed models are able use this information to partition variance between different levels of the temporal hierarchy, such as bars and performances, providing a more powerful analysis than traditional statistical methods, such as multiple regression and ANOVA, which are limited to a single level of the temporal hierarchy. Classical metrics of variability, such SD and IRQ, disregard potentially valuable information about each performance through amalgamation before the data are entered into the inferential analysis. By retaining this information, our stability metric makes it possible to ask new kinds of questions about the roles of stability and flexibility in performance.

**References**

Efron, B., & Tibshirani, R. J. (1993). *An introduction to the bootstrap*. New York: Chapman & Hall, software.

Repp, B. H. (1995). Expressive timing in Schumann’s ‘‘Träumerei:’’An analysis of performances by graduate student pianists. *The Journal of the Acoustical Society of America*, 98(5), 2413-2427.

Van Vugt, F. T., Jabusch, H. C., & Altenmüller, E. (2012). Fingers phrase music differently: trial-to-trial variability in piano scale playing and auditory perception reveal motor chunking. *Frontiers in psychology*, *3*, 495.
